# 22q11.2 Deletion Syndrome Diagnosed 47 Years After Surgery for Tetralogy of Fallot

**DOI:** 10.7759/cureus.48206

**Published:** 2023-11-03

**Authors:** Yuko Harada, Yasuhiko Kanazawa, Tetsuya Tobaru, Kenji Wada, Shuichiro Takanashi

**Affiliations:** 1 Cardiology, Kawasaki Municipal Ida Hospital, Kawasaki, JPN; 2 Diabetes and Endocrinology, Kawasaki Municipal Ida Hospital, Kawasaki, JPN; 3 Cardiology, Kawasaki Saiwai Hospital, Kawasaki, JPN; 4 Cardiovascular Surgery, Kawasaki Saiwai Hospital, Kawasaki, JPN

**Keywords:** obstructive sleep apnea syndrome (osas), hypocalcemia, tetralogy of fallot, digeorge syndrome, 22q11.2 deletion syndrome

## Abstract

A 51-year-old man presented with severe hydrocele testis, dyspnea on exertion, and systemic edema. He had a history of surgery for tetralogy of Fallot (TOF). On the second day of admission, he presented with severe nose bleeding followed by CO_2 _narcosis. Blood gas analysis revealed an extremely low level of Ca^2+^. An echocardiogram revealed an excessively enlarged right ventricle and severe pulmonary valve regurgitation (PR). Hypocalcemia, history of TOF, and characteristic facial features suggested 22q11.2 deletion syndrome, which was confirmed by fluorescence in-situ hybridization (FISH) chromosome test. Open heart redo-surgery was performed for severe PR. The surgery revealed a severely hypoplastic pulmonary valve, which is characteristic of 22q11.2 deletion syndrome. 22q11.2 syndrome thus could be overlooked until age over 50 and therefore become critical.

## Introduction

22q11.2 deletion syndrome is the second most common cause of developmental delay and major congenital heart disease after Down syndrome, with an estimated prevalence of 1 in 4000 live births [1-4]. Clinical features may vary depending on the age of the patient such as developmental disabilities, learning disabilities, conotruncal cardiac anomalies, palatal defects, nasal regurgitation, hypernasal speech, behavioral problems, psychiatric illness, immunodeficiency, hypocalcemia, and characteristic facial features [1]. Because of the significant variability of expression, especially in the absence of classic findings, the diagnosis may be missed [1,5].

This syndrome was historically known as DiGeorge syndrome, which was described by Angelo DiGeorge in 1968 in a series of patients with absent thymic tissue and parathyroid glands [6,7]. Abnormal development of the third and fourth pouches leads to the hallmark features including cardiac anomalies, hypoparathyroidism, and thymic hypoplasia or aplasia [6]. The median age at diagnosis is 360 days, although there have been instances of diagnosis in adulthood [8].

Surgical techniques have advanced in recent years extending the lifespan of congenital heart disease. The transition of medical care from childhood to adulthood could be difficult at times. Here, we report a case of 22q11.2 deletion syndrome who dropped out of physicians’ follow-up in his 20s and was therefore underdiagnosed; he presented with critical heart failure in middle age.

Written consent for this research was obtained from the patient and his family.

## Case presentation

A 51-year-old man presented with severe hydrocele testis and visited the local urologist. His hydrocele was extremely severe and the patient had difficulty in urination. The testis was swollen to 5 cm in diameter and the urethra was narrowed. The patient was referred to our hospital. Since he presented with systemic edema, especially in the extremities, and dyspnea on exertion, he was referred to the division of cardiology. His voice was very low and raspy, and his family said his voice had been raspy since he was born. He didn’t recall when and how the edema and hydrocele started. His mother recalled that a neighbor pointed out that the patient was out of breath on the stairs two months ago, and the patient started to make a mess after urination a few weeks ago.

The past medical history included surgery for tetralogy of Fallot at the age of four, followed by surgery for cleft palate. He underwent cataract surgery for both eyes at the age of 49. He had no family history of congenital heart disease. His father died of myocardial ischemia at the age of 55, and his older brother died of liver cirrhosis at the age of 49.

The patient’s IQ score in his youth is not known. He has never had an IQ test or any other dementia tests. However, he had been very slow in learning at school and struggled to keep up with lessons. He did not drink alcohol but had been smoking 20 cigarettes/day for 31 years.

**Figure 1:**
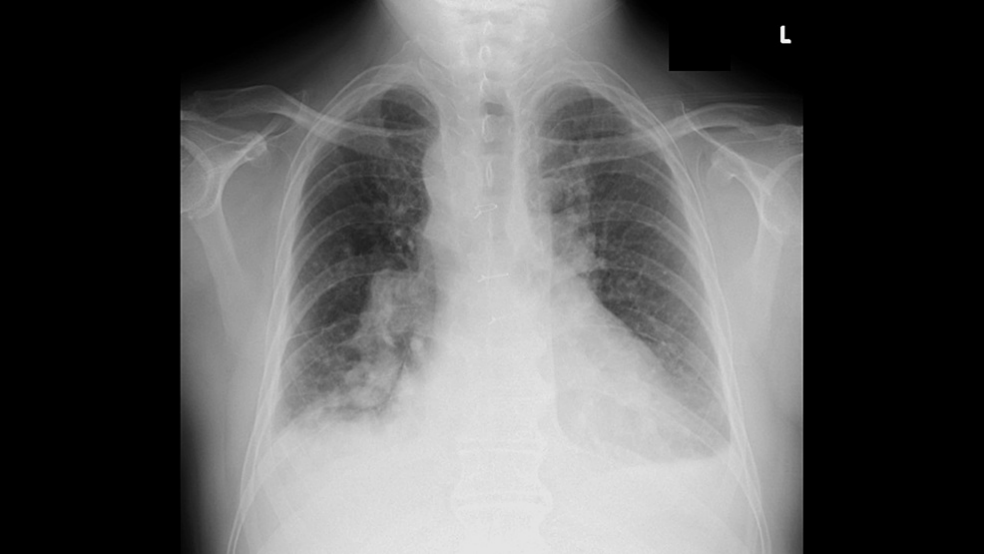
Chest X-ray on admission Cardiomegaly and bilateral pleural effusion are observed.

**Figure 2:**
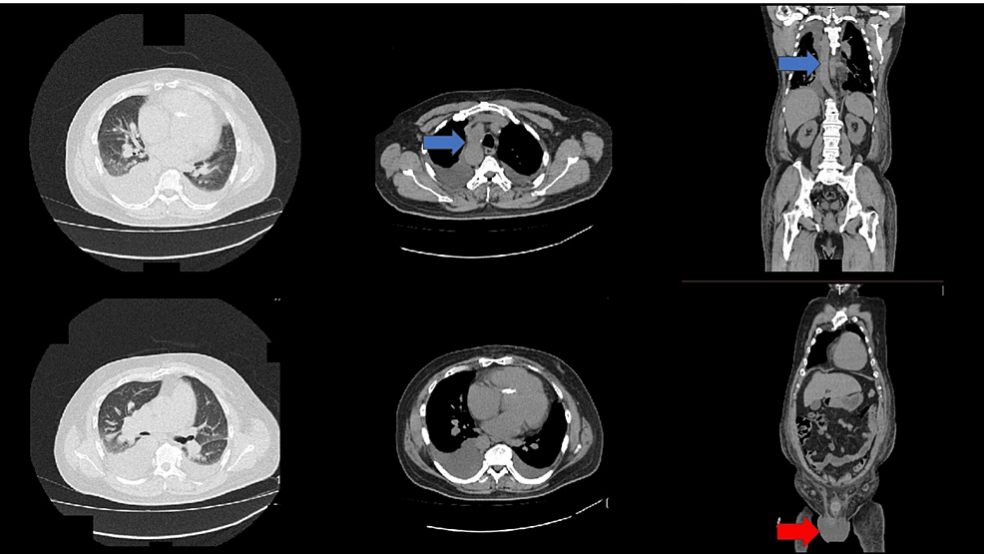
Chest and abdominal CT scan on admission Right-sided aortic arch (blue arrows), cardiomegaly, wall thickening of bronchi, pulmonary congestion, and hydrocele testis (red arrow) are observed.

**Figure 3:**
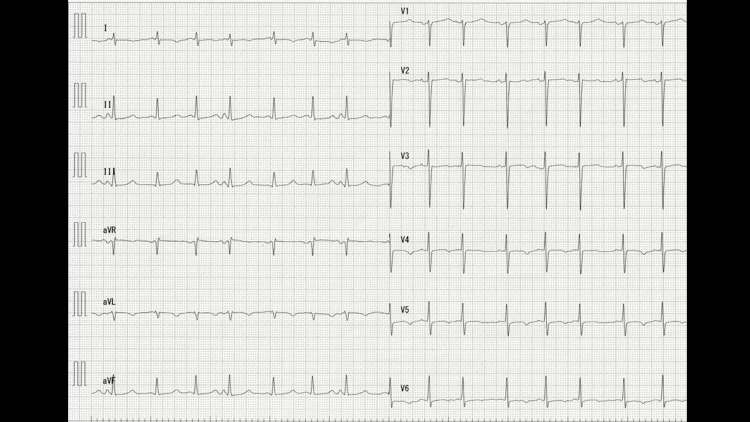
ECG on admission QTc was prolonged to 458 ms.

On admission, the patient was alert without any distress at rest. His body temperature was 36.5℃, and other vital signs were normal. Oxygen saturation (SpO_2_) was 97% in room air. Laboratory data revealed slight anemia of Hb 10.7 g/dL, liver dysfunction of aspartate aminotransferase (AST) 90 IU/L, alanine transaminase (ALT) 48 IU/L, alkaline phosphatase (ALP) 197 IU/L, gamma-glutamyl transpeptidase (γGTP) 69 IU/L, lactate dehydrogenase (LDH) 637 IU/L, elevated C-reactive protein (CRP) level of 5.89 mg/dL, and elevated brain natriuretic peptide (BNP) level of 149.7 pg/mL. Chest X-ray revealed cardiomegaly and bilateral pleural effusion (Figure 1). The chest CT scan revealed a right-sided aortic arch, cardiomegaly, wall thickening of bronchi, and pulmonary congestion (Figure 2). ECG revealed QT prolongation of QTc 458 ms (Figure 3).

The diagnosis on admission was cardiac failure. As the patient had difficulty in urination, a Foley catheter was inserted, which was very difficult due to severe hydrocele. Furosemide 40 mg/day was administered intravenously. Apnea of 7-10 seconds was observed when he fell asleep. Sleep apnea syndrome (SAS) was suspected due to obesity and systemic edema.

**Figure 4:**
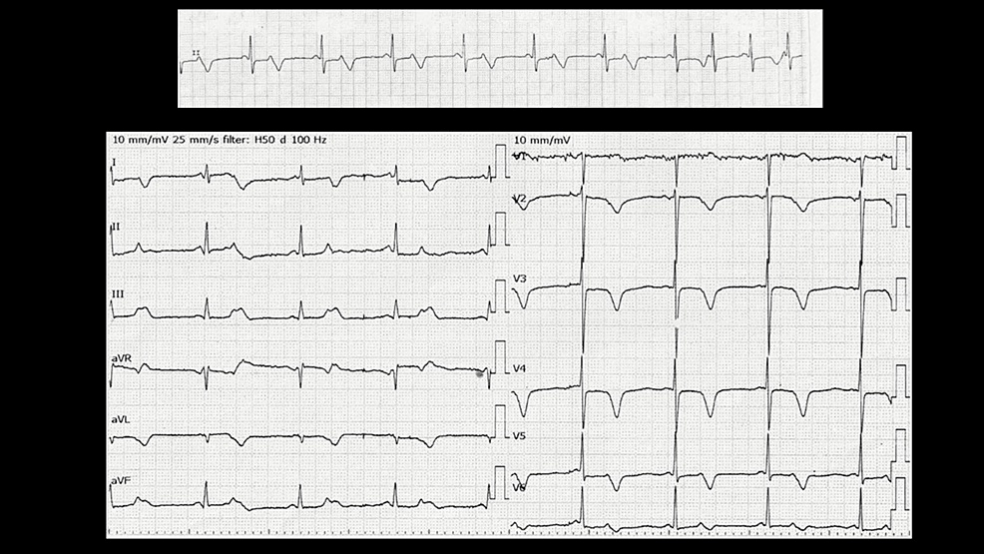
Monitor ECG (top) and 12-lead ECG (bottom) on the third day of admission Multiple blocked APCs are observed. APCs: atrial premature complexes

**Figure 5:**
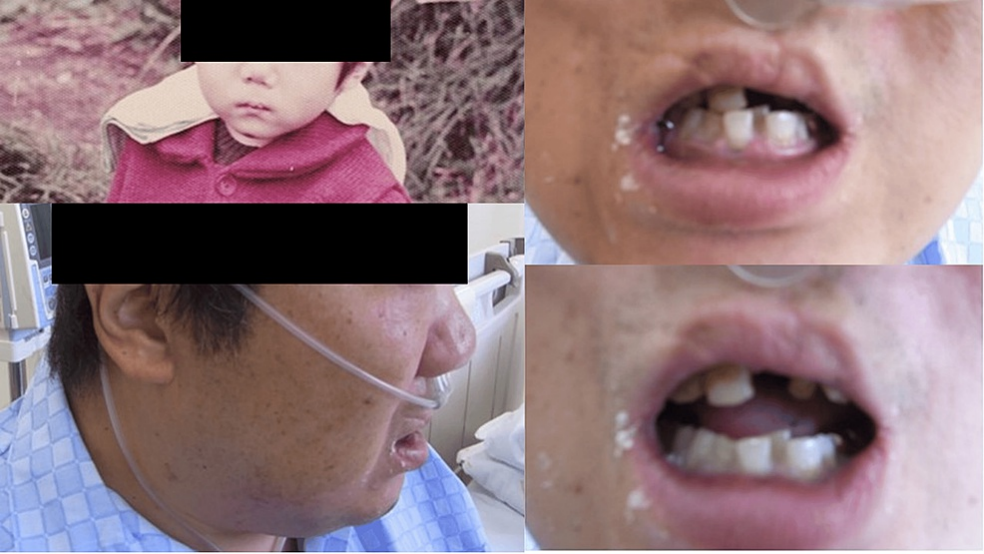
Characteristic facial appearance Left top: The patient in childhood. The patient has low-set ears and a small mouth. Left bottom: Current face of the patient. A small head, low nasal bridge, small jaw, and small mouth are observed. Right top and bottom: The patient’s mouth. Malalignment of teeth is observed.

On the third day of admission, the patient presented with severe nasal bleeding followed by a coma. Blood gas analysis (BGA) revealed pH 7.31, partial pressure of carbon dioxide (PCO2) 99.7 mmHg, partial pressure of oxygen (pO2) 82 mmHg, bicarbonate (HCO3-) 48.7 mmol/L, base excess (BE) 17.5 mmol/L, which confirmed the diagnosis of CO2 narcosis. BGA also revealed extremely low Ca2+ of 0.56 mmol/L (normal range 1.15-1.29). Serum calcium level was 4.7 mg/dL, and total serum calcium level corrected for albumin level was 5.6 mg/dL (normal 8.8-10.5). Monitor ECG revealed bradycardia of 50 bpm with multiple blocked atrial premature complexes (APCs) (Figure 4). Calcium gluconate hydrate 425 mg/day was administered intravenously. Tracheal intubation was difficult due to the patient’s small mouth and small jaws (Figure 5). Non-invasive positive pressure ventilation (NPPV) was performed and the patient became alert within 24 hours. Oral calcium lactate 3 g/day and eldecalcitol 0.75 μg/day were prescribed. The intact parathyroid hormone (PTH) level was 16 pg/mL (normal 10-65), which confirmed the diagnosis of primary hypoparathyroidism.

The patient had been alert and stable for the days. The serum calcium level corrected for albumin level increased to 6.6 mg/dL on the eighth day of admission. The blocked APCs disappeared on monitor ECG. There was a history of TOF, cleft palate, hypocalcemia, and a small mouth associated with 22q11.2 deletion syndrome (DiGeorge syndrome). Fluorescence in-situ hybridization (FISH) of the chromosome revealed deletion of 22q11.2.

**Figure 6:**
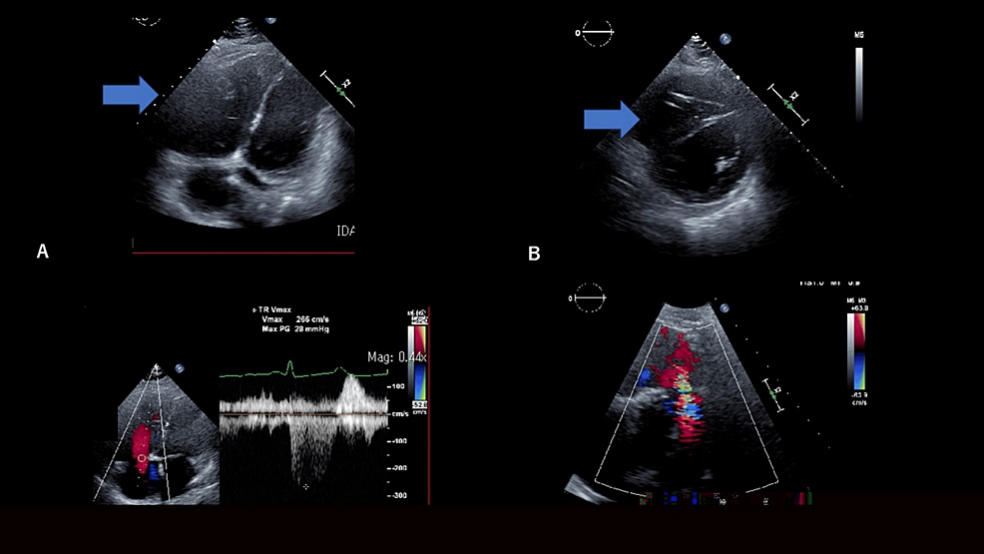
Echocardiography on the seventh day of admission The ejection fraction (EF) was 61.1%. A: Apex four-chamber view. The right ventricle is enlarged (blue arrow). B: Short axis view. The right ventricle is compressing the septum (blue arrow). C: Moderate TR is observed. D: Severe PR is observed. Pressure half-time (PHT) was 83 msec.

The hydrocele and systemic edema were remarkably resolved with furosemide, and the patient’s body weight decreased by 15 kg in seven days (on admission, the patient's weight was 94 kg and his height was 162 cm). Echocardiography revealed an excessively enlarged right ventricle, moderate tricuspid regurgitation (TR), and severe pulmonary valve regurgitation (PR) (Figure 6). Tricuspid annular plane systolic excursion (TAPSE) was 15 mm, S’ was 8.8 cm/sec, and right ventricular fractional area change (RVFAC) was 38%, which indicated that the contractile function of the right ventricle was decreased. These findings confirmed the diagnosis of severe right ventricle failure due to severe PR.

On the eighth day of admission, the patient presented with severe nasal bleeding followed by coma again. His coagulation profile was prothrombin time (PT) 62.2 sec, activated partial thromboplastin time (APTT) 42.0 sec, and platelet count was 16.8x10^4^/μL. BGA revealed pH 7.20, pCO2 138.7 mmHg, pO2 86 mmHg, HCO3- 52.0 mmol/L, and BE 18.8 mmol/L, which confirmed CO2 narcosis. Tracheal intubation was performed for starting synchronized intermittent mandatory ventilation (SIMV). The patient was transferred to another hospital for cardiac surgery.

**Figure 7:**
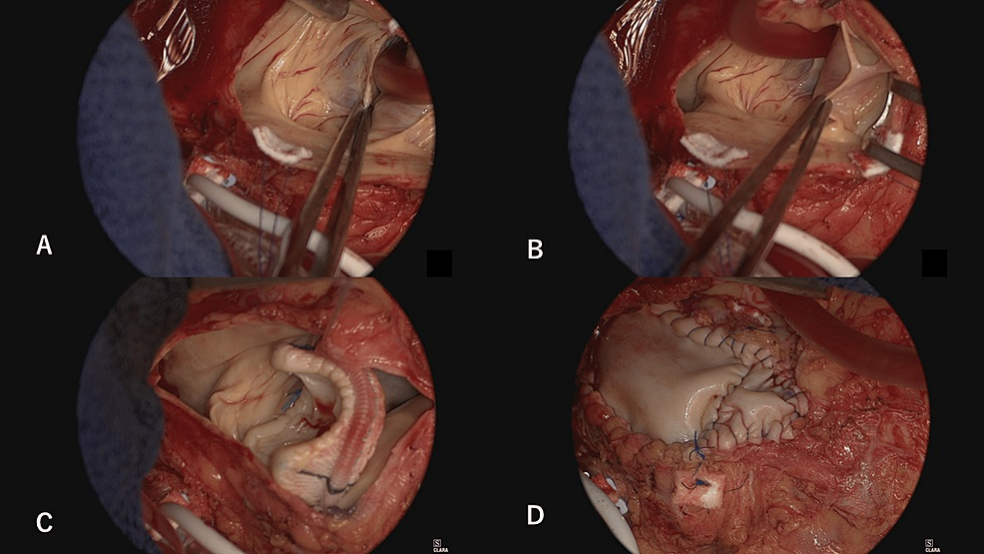
Open-heart surgery A: Left semilunar valve of the pulmonary artery. Among the three cusps, only the left cusp was preserved. B: Anterior semilunar valve of the pulmonary artery was severely hypoplastic. Only a bridging structure was observed. The right semilunar valve was aplastic. C: Installation of an artificial pulmonary valve named the Epic supra. An incision was made into the RVOT on the rear side of the valve, and a mattress suture was performed to fix the valve. D: Patch enlargement was performed on the front side of the valve. RVOT: right ventricular outflow tract

Upon cardiac surgery, the diagnosis was PR due to hypoplastic pulmonary valve, tricuspid valve regurgitant (TR) due to post-leaflet (PL) prolapse, and post-TOF intracardiac repair, which was probably patch-enlargement of the right ventricular outflow tract (RVOT) and ventricular septal defect (VSD) closure. Pulmonary valve replacement and tricuspid valve plasty with plication of PL were performed (Figure 7). The surgeries were successful and the patient was discharged.

## Discussion

22q11.2 deletion syndrome is found in 15% of TOF [9,10]. The FISH chromosome test is now available in many facilities so the diagnosis is much easier than before. However, patients with mild symptoms could be misdiagnosed unless FISH is routinely performed. Patients who survived decades after TOF-intracardiac repair could easily drop out from cardiological check-ups because pediatricians do not treat adult patients of middle age.

In the present case, severe hypocalcemia led to the diagnosis of DiGeorge syndrome. The patient had never taken a chromosome test before, and his family did not recall any suggestion by doctors regarding chromosome abnormalities. The patient was treated by a pediatric cardiologist until his 20s and thereafter had standard adult health check-ups every year. It is not known if his QT prolongation was detected by a health check-up. Symptoms of hypocalcemia are sometimes not remarkable so it could be overlooked. Bilateral cataracts at the age of 49 could have been caused by hypocalcemia, but it was not pointed out. It is not known when and how his edema started, and he was not aware that his health had deteriorated. 22q11.2 deletion syndrome is associated with high rates of schizophrenia, other psychiatric conditions, and difficulty in social skills [11,12]. Such patients could easily drop out from routine medical check-ups.

Open-heart surgery revealed a hypoplastic pulmonary valve, which is a well-known feature of 22q11.2 deletion syndrome [13]. The patient’s severe right heart failure was probably caused by severe PR due to the hypoplastic pulmonary valve. Forty-seven years ago, his hypoplastic pulmonary valve may not have been recognized during TOF-intracardiac repair at the age of 4. If the patient had been undergoing routine UCG, PR would have been detected at an earlier stage.

Long-term outcomes of 22q11.2 deletion syndrome are not known, as the data are limited. However, some researchers have reported that 22q11.2 deletion syndrome is associated with increased mortality (all-cause mortality and cardiac mortality) in adults with TOF [14,15]. Lifelong monitoring is essential for post-surgery TOF patients, desirably with chromosome FISH testing to detect 22q11.2 deletion if any suspicious symptoms appear during follow-up.

The patient’s condition deteriorated every time after nasal bleeding. Severe nasal bleeding had caused airway obstruction leading to CO2 narcosis. Thrombocytopenia is a well-known feature of 22q11.2 deletion syndrome, but the patient’s platelet count was normal. As he had midface hypoplasia probably due to 22q11.2 deletion syndrome, sputum suction may have triggered nasal bleeding. Midface hypoplasia is also known to be at increased risk for obstructive sleep apnea syndrome (OSAS) [16]. The patient’s severe SAS may have been originally caused by midface hypoplasia and worsened by obesity and edema. If his SAS had been recognized earlier, the event of CO2 narcosis could have been prevented.

## Conclusions

22q11.2 deletion syndrome was initially diagnosed 47 years after surgery for TOF due to severe symptomatic hypocalcemia. The patient also suffered from life-threatening right heart failure due to severe PR caused by 22q11.2 deletion syndrome. If the patient had been diagnosed earlier, he would have been treated with diuretics, calcium lactate, and eldecalcitol, and his prognosis could have been better.
